# Metabolomics and Transcriptomics Analysis of Pollen Germination Response to Low-Temperature in Pitaya (*Hylocereus polyrhizus*)

**DOI:** 10.3389/fpls.2022.866588

**Published:** 2022-05-12

**Authors:** Hong-fen Dai, Biao Jiang, Jun-sheng Zhao, Jun-cheng Li, Qing-ming Sun

**Affiliations:** ^1^Key Laboratory of South Subtropical Fruit Biology and Genetic Resource Utilization (Ministry of Agriculture and Rural Affairs), Guangdong Province Key Laboratory of Tropical and Subtropical Fruit Tree Research, Institute of Fruit Tree Research, Guangdong Academy of Agricultural Sciences, Guangzhou, China; ^2^Vegetable Research Institute, Guangdong Academy of Agricultural Sciences, Guangzhou, China; ^3^Center of Agricultural Science and Technology Promotion, Maoming, China

**Keywords:** pitaya, low-temperature storage, transcriptomics, metabolomics, *in vitro* pollen germination

## Abstract

Cross-pollination can improve the percentage of fruit set and fruit weight for most red flesh varieties in pitaya. The technology of pollen storage was very important for successful cross-pollination. However, till present, the technology of pollen storage is unsatisfactory in pitaya production. In this study, pitaya pollen stored at low temperature was taken as the research object, and its physicochemical indexes, metabolomics, and transcriptomics were studied. The results showed that *in vitro* pollen germination rate decreased significantly with the increase in storage time. Soluble sugar and soluble protein content of pollen peaked on the first day of storage, whereas its relative conductivity, and manlondialdehyde (MDA) and proline contents increased gradually during storage. At the same time, the antioxidant enzyme system of pollen was also affected. Superoxide dismutase (SOD) activity decreased, while the activities of catalase (CAT) and peroxidase (POD) increased and superoxide anion generation rate increased gradually during storage. According to the metabolomics results, amino acid, peptide, nucleotide, plant hormone, terpene, alcohol, phenol, flavonoid, sterol, vitamin, ester, sphingolipid, and ketone contents increased significantly during storage, whereas flavonoid and pigment contents declined gradually. During pollen storage, the gene expressions related to carbohydrate metabolism, protein metabolism, acid and lipid metabolism, sterol metabolism, plant hormone metabolism, and signal transductions were significantly downregulated. With KEGG pathway analysis, isoquinoline alkaloid biosynthesis, tyrosine metabolism, alanine, aspartate, and glutamate metabolism of pollen were affected significantly during low-temperature storage. Correlation analysis showed that the gene expression patterns of *HuRP2, HuUPL1*, and *HuAAT2* had significant effects on pollen germination. D-arabinose 5-phosphate and myricetin were positively correlated with pollen germination rate, which was valuable for studying preservation agents. In this study, the changes in pollen during low-temperature storage were described from the level of metabolites and genes, which could provide theoretical support for the research and development of pollen long-term storage technology in pitaya.

## Introduction

Pitaya (*Hylocereus*) is a new fruit crop, which originates from Latin America (Fan et al., 2018). With its attractive pulp color and nutritional benefits, pitaya is widely grown in tropical and subtropical regions (Matan et al., 2015). According to pulp color, pitaya could be divided into white, yellow, and red flesh varieties (Grimaldo-Juárez et al., 2007). Among these varieties, consumers pay more attention to the red flesh varieties because of their high antioxidant activity, which benefits human health (Wu et al., 2006). However, at present, most of the red flesh varieties are completely or partially self-incompatible. Under the condition of natural pollination, flowers and fruits fall severely, or even if they successfully set fruits, the fruits are small, which significantly affects their economic value. Artificial pollination using pollen from other varieties was helpful to increase the fruit setting rate and single fruit weight and improve the fruit quality and flavor (Tran et al., 2015).

However, in production, due to weather and other reasons, pollinated varieties and cultivated varieties sometimes have different flowering dates, leading to pollination failure. At this time, it is very important to preserve pollen *in vitro* for artificial pollination. The success rate of artificial pollination and fruit set is closely related to pollen viability (Rosell et al., 2006; Mendes et al., 2012). Therefore, pollen viability must be maintained during *in vitro* preservation. Macha et al. (2006) found that the viability of pollen is lost under too dry or wet conditions, and fresh pollen can be stored in a vial at 5°C for 8 days. They also found that the pollen germination and tube elongation were reduced after being stored at −20 or −30°C for 15 days, and the pollen did not germinate after being stored in acetone and ethyl acetate. The conditions required for *in vitro* pollen preservation of pitaya are different from those of other species (Khan and Perveen, 2014; Pham et al., 2015; Muradolu et al., 2017), and the studies on pollen storage in pitaya are limited.

With the development and popularization of omics technology, an increasing number of studies focusing on pollen grain function, including transcriptomics (Keller et al., 2018), proteomics (Chaturvedi et al., 2016; Keller et al., 2018), and metabolomics (Fragallah et al., 2018; Paupière et al., 2019), have been conducted in recent years. With the application of transcriptomics technology, Xu et al. (2021) found that the abnormal growth of the *Clonorchis sinensis* pollen tube caused by NO-induced by low temperature could be due to the differential expression of miR5721, miR5819, and miR1429-3p, and novel_mir_18 regulated their target genes related to pollen tube growth, resulting in changes in insufficient energy, cell wall organization, and ion distribution for pollen tube growth, thus inhibiting polar growth of *C. sinensis* pollen tube tips after low-temperature treatment. Fragallah et al. (2018) focused on different germination abilities from two clones of Chinese fir [*Cunningham ia lanceolata* (Lamb.) Hook]. The results showed that there were significant differences in metabolites between different clones at different stages of germination, and the expression of metabolites in the early stage of germination differed from that in the late stage of pollen tube growth. Due to the inhibition of the expressions of anther cell wall invertase and monosaccharide transporter genes, starch accumulation in mature pollen grains and sugar accumulation in anther are blocked simultaneously, which leads to a decrease in pollen bank strength (Koonjul et al., 2005; Oliver et al., 2005; Ji et al., 2011). Furthermore, Wada et al. (2020) indicated that picoPPESI-MS analysis can efficiently identify the metabolites in intact single pollen.

Therefore, this research observes the physical and chemical changes of pollen during low-temperature storage in pitaya, combined with metabolomics and transcriptomics. The purpose was to find metabolites and genes that are closely related to pollen low-temperature storage in order to provide theoretical support for the research and development of pollen long-term storage technology in pitaya.

## Materials and Methods

### Plant Materials and Treatments

The pollen grains were derived from a red-skin purplish flesh-type pitaya (*Hylocereus polyrhizus*). After 21:00 on a clear night, 300 flowers of similar size that opened that night were randomly chosen from the field. Pollen grains were collected into clean paper cups by shaking flowers manually, mixed thoroughly, and brought back to the laboratory with a 100 mesh sieve to filter impurities, including worms, anthers, and petal fragments. The pollen grains were placed at 4°C and were sampled on days 0, 1, 2, and 3, respectively. And then, the pollen grains were divided into several parts and stored at −80°C for future experiments.

### Measurement of Pollen Germination

The pollen germination was determined as described by Mustad et al. (2006). For *in vitro* germination, 0.1 g of pollen grain was placed in a 1-ml liquid medium and incubated at a temperature incubator (30°C and RH80%) for 20 h, and the germination rate was recorded. The components of the liquid medium were as follows: 30% sucrose +500 mg · L^−1^ H_3_BO_3_.

The observation was conducted in the open field using a ZEISS Axio Scope A1 fluorescence microscope (200 ×). Pollen germination was determined by the criterion that the length of the pollen tube was greater than or equal to the diameter of pollen grains. In each sample, three fields were observed, and each field included no less than 30 pollen grains. The photographs were taken using an AxioCamHRc camera and Axio Vision Rel 4.8 microphotography software. Germination rate = number of germination pollen/total number of pollen grains × 100%. Three replicates were measured.

### Measurement of Soluble Sugar and Protein

According to the manufacturer's instructions, measurement of soluble sugar was performed as described in the test kit (D799391-0050, Sangon Biotech Co., Ltd, Shanghai, China). Three replicates were measured.

Measurement of soluble protein: A pollen grain sample of 0.1 g was weighed and ground with 5 ml distilled water for extraction. Of the extract, 1 ml was absorbed and placed in the test tube, and 5 ml of Coomassie Brilliant Blue G-250 solution was added. After 2 min, the absorbance value was measured at 595 nm, and the protein content was calculated through the standard curve, which was denoted as X. The standard curve was drawn with the protein concentration as the abscissa and the absorption value as the ordinate. The soluble protein content of the sample (mg/g) = (C^*^V_T_)/(${\text{V}}_{1}^{*}$FW^*^1,000), where C is the value of the standard curve (μg), V_T_ is the total volume of the extract (ml), FW is the fresh weight of the sample (g), and V_1_ is the amount of sample added in the determination (ml). Three replicates were measured.

### Measurement of Relative Conductivity and Content of MDA

The relative conductivity was determined as described by Zhao et al. (2020). Three replicates were measured. The content of MDA was measured as described in the manufacturer's instructions of the test kit (D799761-0050, Sangon Biotech Co., Ltd., Shanghai, China). A pollen grain tissue of 0.1 g was weighed, and three replicates were measured.

### Measurement of the Content of Proline

A pollen grain tissue of 0.1 g was weighed. The content of proline was measured as described in the manufacturer's instructions of the test kit (D799575-0050, Sangon Biotech Co., Ltd., Shanghai, China). Three replicates were measured.

### Measurement of Activity of Antioxidative Enzymes

A pollen grain of 0.1 g was weighed. In an ice bath, 1 ml of extract liquid was added and homogenized. Following centrifugation at 8,000 *g* at 4°C for 10 min, the supernatant was taken, which was the sample extract, and placed on ice for testing. Three replicates were measured.

Measurement of activity of SOD was performed as described in the manufacturer's instructions of the test kit (D799593-0050, Sangon Biotech Co., Ltd, Shanghai, China).

Measurement of activity of CAT was performed as described in the manufacturer's instructions of the test kit (D799597-0050, Sangon Biotech Co., Ltd, Shanghai, China).

Measurement of activity of POD was performed as described in the manufacturer's instructions of the test kit (D799591-0050, Sangon Biotech Co., Ltd, Shanghai, China).

### Measurement of Superoxide Anion Generation Rate

According to the manufacturer's instructions, a 0.1 g pollen grain sample was weighed. The measurement of the superoxide anion generation rate was performed as described in the test kit (D799771-0050, Sangon Biotech Co., Ltd., Shanghai, China). Three replicates were measured.

### Metabolomics Analysis

Sample extraction: In a 2 ml EP tube, 200 mg (±1%) of the sample was accurately weighed, 0.6 ml of 2-chlorophenylalanine (4 ppm) methanol (−20°C) was added, and the sample was vortexed for 30 sec. The 100 mg glass beads were added, and the samples were put into a Tissue Lysis II tissue grinding machine. The samples were ground at 25 Hz for 60 s, treated with ultrasound at room temperature for 15 min, centrifuged at 25°C for 10 min at 12,000 rpm, and the supernatant was filtered through a 0.22-μm membrane to obtain the prepared samples for LC-MS. From each sample, 20 μl was taken to the quality control (QC) samples^*^ (these QC samples were used to monitor deviations of the analytical results from these pool mixtures and compared them to the errors caused by the analytical instrument itself). The rest of the samples were used for LC-MS detection. Five replicates were measured.

The chromatographic conditions: Chromatographic separation was accomplished in a Thermo vanquish system equipped with an ACQUITY UPLC^®^ HSS T3 (150 × 2.1 mm, 1.8 μm, Waters) column maintained at 40°C. The temperature of the autosampler was 8°C. Gradient elution was carried out at a flow rate of 0.25 ml/min using 0.1% formic acid in water (A_1_) and 0.1% formic acid in acetonitrile (B_1_) or 5 mM ammonium formate in water (A_2_) and acetonitrile (B_2_). After equilibration, 2 μl of each sample was injected. An increasing linear gradient of solvent B (v/v) was used as follows: 0–1 min, 2% B_2_/B_1_; 1–9 min, 2%−50% B_2_/B_1_; 9–12 min, 50%−98% B_2_/B_1_; 12–13.5 min, 98% B_2_/B_1_; 13.5–14 min, 98%−2% B_2_/B_1_; 14–20 min, 2% B_1_-positive model (14–17 min, 2% B_2_-negative model).

Mass spectrometry conditions: The ESI-MSn experiments were executed on the Thermo Q Exactive Plus mass spectrometer with a spray voltage of 3.5 kV and −2.5 kV in positive and negative modes, respectively. The arbitrary units of sheath gas and auxiliary gas were set at 30 and 10, respectively. The capillary temperature was 325°C. The analyzer scanned over a mass range of m/z 81–1,000 for a full scan at a mass resolution of 70,000. Data-dependent acquisition (DDA) MS/MS experiments were performed using an higher energy collision induced dissociation (HCD) scan. The normalized collision energy was 30 eV. Dynamic exclusion was implemented to remove some unnecessary information from MS/MS spectra.

### Transcriptomics Analysis

RNA degradation and contamination were monitored on 1% agarose gels. RNA purity was checked using the NanoPhotometer^®^ spectrophotometer (IMPLEN, CA, USA). RNA integrity was assessed using the RNA Nano 6000 Assay Kit of the Agilent Bioanalyzer 2100 system (Agilent Technologies, CA, USA). Three replicates were measured.

A total of 1 μg RNA per sample was used for the RNA sample preparations. The sequencing libraries were generated using NEBNext^®^ Ultra™ RNA Library Prep Kit for Illumina^®^ (NEB, USA) following the manufacturer's recommendations. Briefly, mRNA was purified from total RNA using poly-T oligo-attached magnetic beads. Fragmentation was carried out using divalent cations under elevated temperature in the NEB Next First Strand Synthesis Reaction Buffer (5X). The library fragments were purified using the AMPure XP system (Beckman Coulter, Beverly, USA). Finally, PCR products were purified (AMPure XP system) and the library quality was assessed on the Agilent Bioanalyzer 2100 system.

For sequencing, the Illumina Hiseq X Ten platform and 150 bp paired-end read were used.

Data analysis included quality control, *de novo* assembly, functional annotation of the assembled Unigenes, quantification of gene expression level, differential expression analysis, KEGG pathway enrichment analysis, and correlation analysis.

### Q-PCR Validation

To validate the transcriptomics data, we evaluated 27 significantly changed genes *via* Q-PCR. Total RNA was extracted from pollen using a Plant RNA Kit (R6827-01, Omega Biotek, USA) according to the manufacturer's instructions. RNA was treated with the PrimeScript RT reagent Kit (RR037A, Takara, Japan) to reverse transcription RNA. As shown in Table 1, primers were then designed for the 27 genes being examined, and quantitative RT-PCR, transcriptional normalization, and relative quantification were performed as previously described using three biological replicates with the SYBR Premix Ex TaqKit (RR420A, Takara, Japan).

**Table 1:** Sequences of the specific primers for the genes tested in real-time PCR experiments.

**Gene_ID**	**Forward**	**Reverse**
Cluster-11144.10123	GTGTCCTCCTACCCTGAATGGAAATAC	AACCGCACCTTCTATGCTGATTGG
Cluster-11144.10188	GAGTTGAGCCACGACTTGATCCTG	ACGGTATCAGCATTGGAAGCCTTG
Cluster-11144.10198	GAGACGGTGAATACTACATGGTGACTG	TTGAAGCACAGCATGGCGAAGG
Cluster-11144.10237	TGTCAACTTCTTCAGGAGCCTCAATG	CTCTTCCCTTCTGCCTCTCACCTC
Cluster-11144.10281	AAGTATGCTGCTGGAGGAAACGAAG	TCCTTCTCCTTGTCCTTGTCCTTCTC
Cluster-11144.10321	CCACAAGATGCCGAGTCCTTCAC	AACCAGCAACGGATTCACTAGGC
Cluster-11144.10390	CACAGCAGATGGAGTTCCGTATCAC	GTTCCAGTTTCCTTGAAGCGTTGTTC
Cluster-11144.10421	CCTTCCCAAAGCGAGTACCACATC	ACATTCCTGTTCACCGTGTCTAGTTG
Cluster-11144.10442	GAAACTGAGAACGAGGACAAGACTGAG	GGAGACACCAGATTCATCAACAGGAG
Cluster-11144.10521	CTCAAGGTCGTTCACTGCGTACTC	AAGGATGTCATTCAAGGTGGCTAAGG
Cluster-11144.10545	CTCAATGAAGGGTCGTCAGTTCTACAG	GACAGTTCTGGTACACCGCTTGG
Cluster-11144.10561	GACCGCCGAGCAGGAAGATATAATG	GGCAAATGTGGAAATAGGCAGAATGAG
Cluster-11144.10574	GAAACGGAAGCAAGGGCAGGAG	AGAGGAGGTTGAGATTGAGGAGGATG
Cluster-11144.10611	GGTTGGTCTTCTCTTCCCTTCTTGTC	CACCACCACCTATTTCCCTCTTTCTC
Cluster-11144.10615	ATTTGCTTCATTTGTTCATCCCAGTGG	CAACTCTGCCAAAGAATCATGCCAAG
Cluster-11144.10630	GTCTTTGCCTGGGTGGGTGTTC	CCTGATCCGAATGCCGTGGTTG
Cluster-11144.10633	GAACCAGCAGAGTGACCAGGAATG	CTCAGGCTCAGGATTTAAGTCCAAGG
Cluster-11144.10637	GCCTCTACCTTTGTGGGAAACTCG	TCTGCCTCGGTGAACTCCATCTC
Cluster-11144.10686	AGAGGTGACCAAGAGGGTAGATGC	CTCCTGACGGCGTGAAGAATGC
Cluster-11144.10688	AACACGATTGTAGTTGGACCAGCAG	GTCAACGCCATCACCCTCACTG
Cluster-11144.10703	TGACCTAGAGCGGACCATAGATTCG	CCTGCCTCGGTGCTTGTATTCTC
Cluster-11144.10714	GGCGATGACAAGGAGCGTGAAG	CTTTGGATCTTGAGGTTGGCATTTCTC
Cluster-11144.10730	GCATATTACGAAGAGGGGAAGATGGC	TGACCTTGACCTTGAACTTGACCTTG
Cluster-11144.10748	TTACCATCTCCAACAACCACATGACTC	GCATCCTCTGAACCAACCCTTTCC
Cluster-11144.10791	TGTGCTCGTAGGCTTGAATCTTATGG	GCAAGAGAAGGTGGACAAGATGAAGG
Cluster-11144.10884	ACCTGATGATGGCTGTTGCTTGG	TATGACTCGCTTGATGTTCACCTTGG
Cluster-11144.9843	ATCCACAGCCTCCTCCAACGAC	GTGGGAGAAGATGGCAGCAAGAC

*The first column indicates the ID information of the Unigene. The sequences of forward and reverse primers are given in the second and third columns*.

### Statistical Analysis

The experiments were completely randomized. The results of the experiments were expressed as the mean values of three biological replicates. The PLS-DA analyses were determined by SMICA version 13.0. The significant differences in the result of each experiment were determined by the independent-samples T-test (p < 0.05) using SPSS version 16.0.

## Results

### Effects of Low-Temperature Storage on Physicochemical Parameters of Pollen in Pitaya

**Figure 1:**
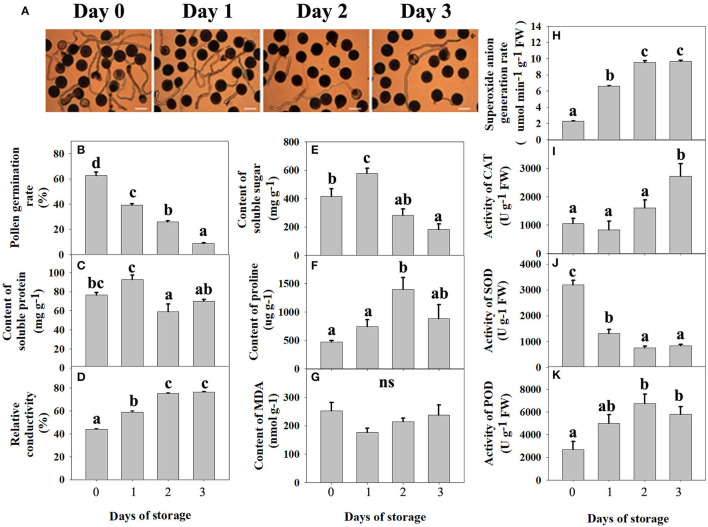
Physical and chemical changes in pitaya pollen during low-temperature storage. **(A)** Microscopic examination of pollen germination from days 0 to 3. The observation was conducted under a microscope (200 ×) in the open field. Three fields were observed in each sample, and three replicates were measured. Bars represent 100 μm. **(B)** *In vitro* pollen germination rate. **(C)** Content of soluble protein. **(D)** Relative conductivity. **(E)** Content of soluble sugar. **(F)** Content of proline. **(G)** Content of manlondialdehyde (MDA). **(H)** Superoxide anion generation rate. **(I)** The activity of catalase (CAT). **(J)** The activity of superoxide dismutase (SOD). **(K)** The activity of peroxidase (POD). In **(B–K)**, each bar was the mean ± standard error of three replications, and bars with different letters were significantly different at *P* < 0.05 using the independent samples t-test.

According to Figures 1A,B, with the increase in storage time, the number of pollen tubes decreased gradually, and the pollen germination rate decreased significantly. The pollen germination rate was 62.7% on day 0 but decreased sharply to 8.7% after 3 days of storage. Both soluble protein (Figure 1C) and soluble sugar (Figure 1E) showed a trend of first increasing and then decreasing during low-temperature storage and reached the maximum value 1 day after storage, the contents of which were 576.49 mg g^−1^ and 92.63 mg g^−1^, respectively. As shown in Figure 1D, relative conductivity increased gradually along with storage, which showed that the pollen cell membrane permeability increased during low-temperature storage. The total MDA content (Figure 1G) decreased on day 1, and then increased, but there was no significant difference during storage. With the increase in storage time, proline gradually accumulated in pollen, reaching a peak value on the second day of storage, with a content of 1,399.9 μg g^−1^, which was three times higher than that on the 0 day of storage (Figure 1F).

As shown in Figure 1H, the superoxide anion generation rate increased significantly during storage time. According to Figure 1J, the activity of superoxide dismutase peaked on day 0 and decreased significantly along with storage. However, the activity of catalase (Figure 1I) and peroxidase (Figure 1K) increased gradually with storage and reached the maximum value on the third and second day of storage, respectively. An imbalance of enzyme activity, including superoxide dismutase, catalase, and peroxidase, eventually leads to an imbalance in the antioxidant system in the pollen grain, leading to the accumulation of large amounts of free radicals.

### Metabolomics Analysis on Pollen Response to Low-Temperature Storage in Pitaya

**Figure 2:**
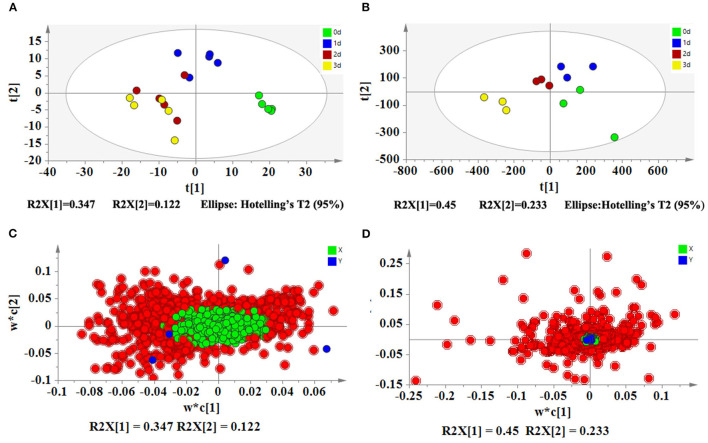
PLS-DA analyses of metabolomics and transcriptomics of pitaya pollen under low-temperature storage. **(A)** PLS-DA score plot of metabolomics. **(B)** PLS-DA score plot of transcriptomics. **(C)** PLS-DA loading plot of metabolomics. **(D)** PLS-DA loading plot of transcriptomics. PLS-DA score plots **(A,B)** show that four groups (days 0, 1, 2, and 3) are located in different regions, indicating that the metabolites and genes are changed significantly during storage. The results of PLS-DA loading plots **(C,D)** show that the important metabolites and genes (VIP>1) are in red.

**Figure 3:**
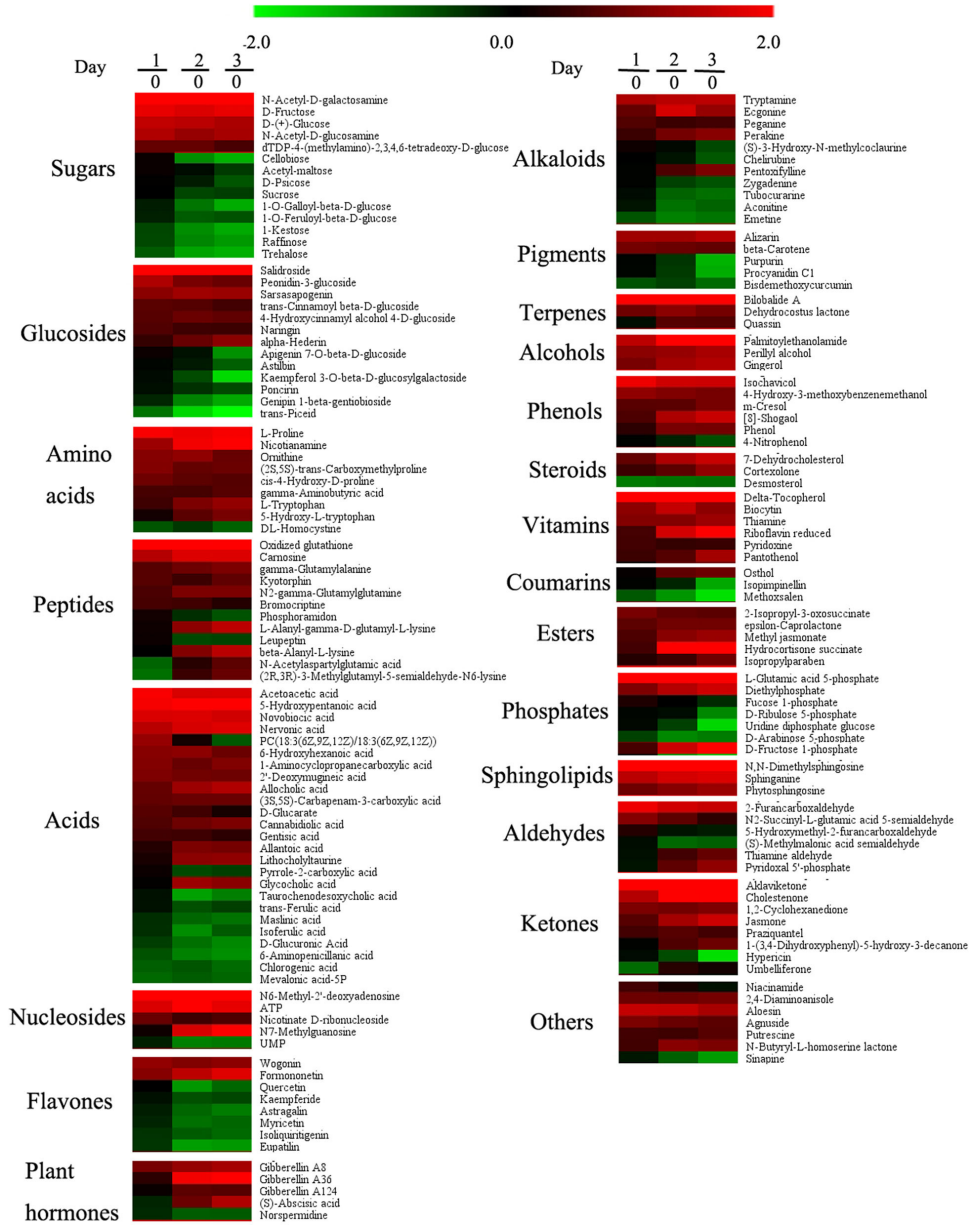
Effects of low temperature on metabolites of pollen in pitaya. Each data point represents a mean (*n* = 5). Samples on days 0, 1, 2, and 3 were used for the experiment and 167 metabolites met the conditions (VIP > 1 and *p* < 0.05) as differentially expressed metabolites.

Based on LC-MS, we used untargeted metabolomics to determine the change of metabolites in pollen during low-temperature storage. The results showed that a total of 1,859 compounds were detected, and 1,216 and 643 compounds were detected by positive and negative ion models, respectively. After data pretreatment, the metabolomics data was analyzed using PLS-DA. According to the score plot in Figure 2A, day 0 samples were distributed in the fourth quadrant, day 1 samples were distributed near the positive half axis of the Y-axis, and day 2 and day 3 samples were distributed in the third quadrant, indicating that there were significant differences in metabolites in pollen from different storage periods. After storage for 2 days, the composition gradually converged. After PLS-DA analysis and ANOVA analysis, metabolites were defined as differential metabolites with VIP >1 and p <0.05 and were specially marked in red in the load diagram (Figure 2C). The differential metabolites were evenly distributed in the regions far from the origin, indicating that they largely contributed to the model. We found a total of 167 different metabolites, including 15 sugars, 13 glycosides, 9 amino acids, 12 peptides, 25 acids, 5 nucleosides, 8 flavonoids, 5 plant hormones, 11 alkaloids, 5 pigments, 3 terpenes, 3 alcohols, 6 phenols, 3 sterols, 6 vitamins, 3 coumarins, 5 esters, 6 phosphates, 3 sphingolipids, 6 aldehydes, 8 ketones, and 7 other compounds (Figure 3).

In sugars and glycosides, D-(+) -glucose, N-acetyl-D-galactosamine, D-fructose, N-acetyl-d-glucosamine, DTDP-4-(methylamino)-2,3,4, 6-tetradeoxy-d-glucose, salidroside, peonidin-3-glucoside, sarsasapogenin, *trans*-cinnamoyl beta-D-glucoside, 4-hydroxycinnamyl, 4-D-glucoside, naringin, and alpha-hederin increased significantly during storage, while the contents of cellobiose, acetyl-maltose, D-psicose, sucrose, apigenin 7-O-beta-D-glucoside, astilbin, kaempferol 3-O-beta-D-glucosylgalactoside, and poncirin were stable at on day 0 and day 1, and decreased markedly on day 2 and day 3, while other sugars and glucisides went down during storage (Figure 3). Most of acid contents increased significantly, and a few decreased during storage, such as taurochenodesoxycholic acid, *trans*-ferulic acid, maslinic acid, isoferulic acid, D-glucuronic acid, 6-aminopenicillanic acid, chlorogenic acid, and mevalonic acid-5P. The decrease in D-glucuronic acid meant that the pollen grain lacked the main component during storage, which affected the pollen germination significantly. According to Figure 3, amino acid, peptide, nucleotide, plant hormone, terpene, alcohol, phenol, sterol, vitamin, ester, sphingolipid, and ketone contents increased significantly, whereas flavone and pigment contents declined gradually in the process of storage.

### Transcriptomics Analyses of Pollen Response to Low-Temperature Storage in Pitaya

**Figure 4:**
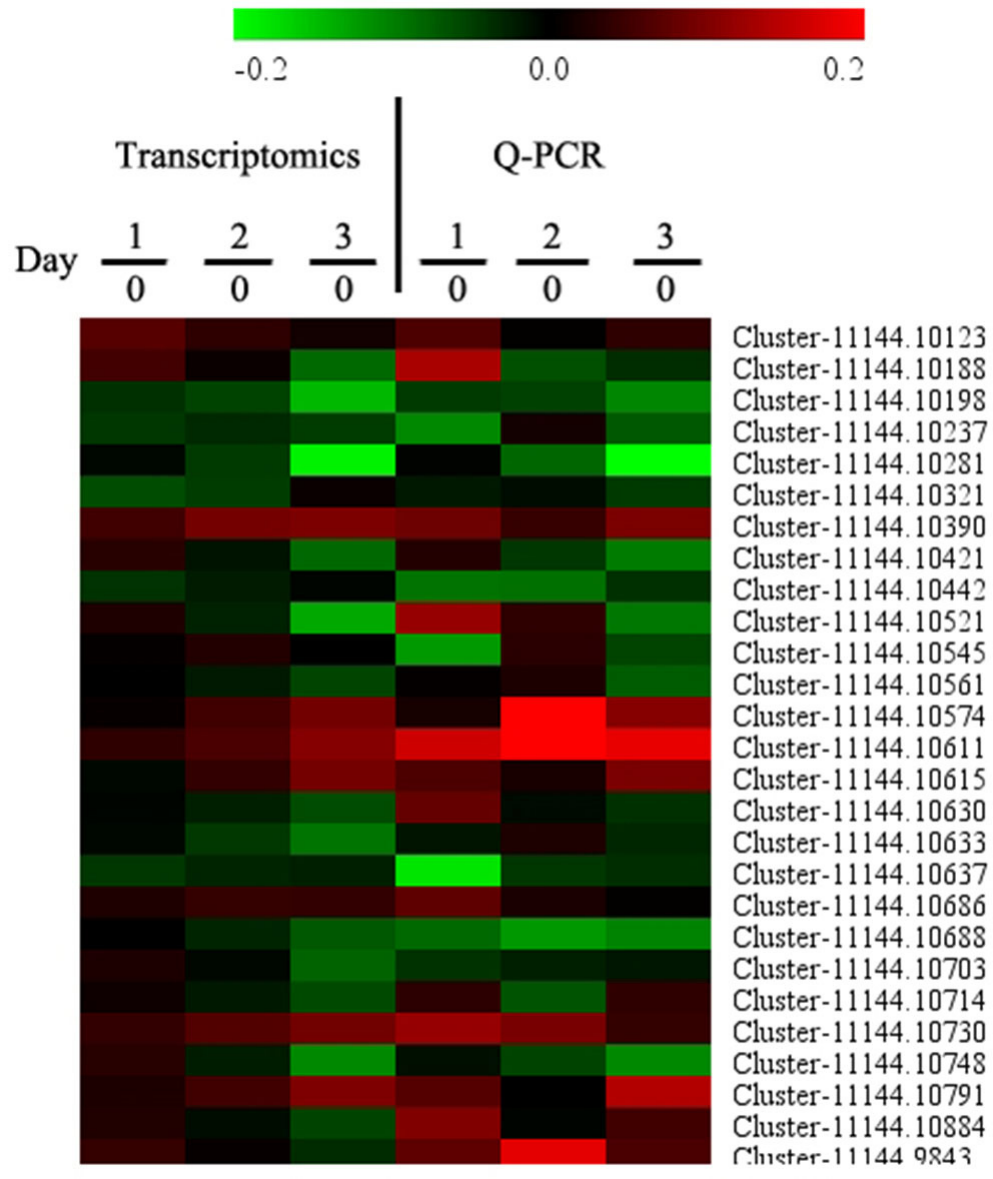
Quantitative real-time PCR (qRT-PCR) analysis of a set of DEGs. Samples on days 0, 1, 2, and 3 were used for Q-PCR conformation experiment. Gene expression on day 0 was set as 1, and the relative expression level was calculated for tested genes. Each data point represents a mean (*n* = 3).

To confirm the transcriptomics data, we evaluated 27 genes *via* qRT-PCR. As shown in Figure 4, there were similar trends between transcriptomics data and qRT-PCR data, indicating that the transcriptomics data were reliable.

**Figure 5:**
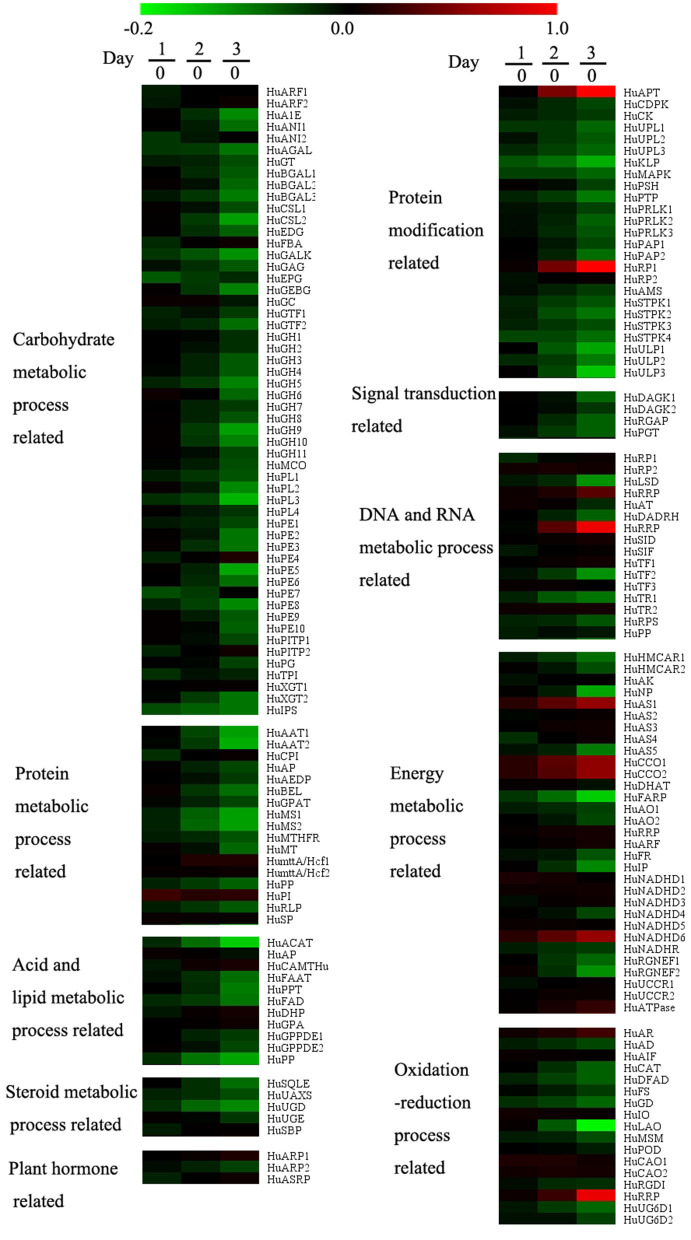
Effect of low temperature on gene expressions of pollen in pitaya. Each data point represents a mean (*n* = 3). Samples on days 0, 1, 2, and 3 were used for the transcriptomics experiment and 174 genes met the conditions (VIP > 1 and *p* < 0.05) as differentially expressed genes.

A total of 14,599 genes were detected in the transcriptomics data. After data pretreatment, we first performed PLS-DA analysis on the transcriptomics data. According to the score plot (Figure 2B), day 0 samples were distributed in the fourth quadrant, day 1 and day 2 samples were distributed near the positive Y-axis, and day 3 samples were distributed in the third quadrant, indicating that there were significant differences in the expression of genes during pollen storage. Combined with ANOVA analysis, 174 genes were defined as differential genes that were matched with VIP >1 and p <0.05 and were specially marked in red in the load diagram (Figure 2D). These genes included carbohydrate metabolism, protein metabolism, acid and lipid metabolism, sterol metabolism, plant hormone metabolism, protein modification, signal transduction, DNA and RNA metabolism, energy metabolism, and oxidation and reduction reaction (Figure 5).

According to the heat map (Figure 5), the genes related to carbohydrate metabolism, protein metabolism, acid and lipid metabolism, sterol metabolism, plant hormone metabolism, and signal transductions were downregulated during storage. The accumulation of sugar was hindered by the inhibition of genes related to carbohydrate metabolism, which led to the disorder of energy metabolism. Genes related to DNA and RNA metabolism, energy metabolism, and REDOX reaction, only *HuAPT, HuRP1, HuRRP, HuAS1, HuCCO1, HuCCO2, HuNADHD6*, and *HuRRP* were upregulated during storage.

### Pathway Analysis of Pollen Response to Low-Temperature in Pitaya

**Figure 6:**
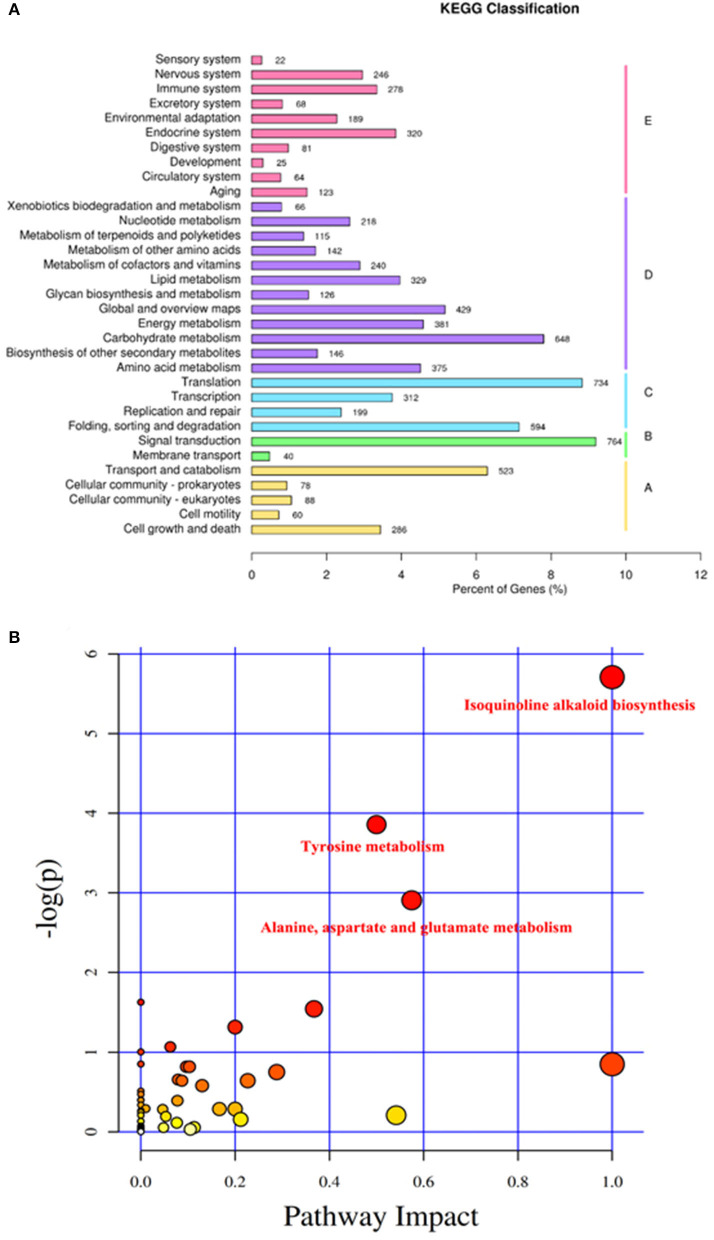
KEGG classification and key KEGG pathway analysis of transcriptomics. **(A)** KEGG classification of transcriptomics. Genes were divided into five branches according to the KEGG pathways involved in I, Cellular processes; II, Environmental information processing; III, Genetic information processing; IV, Metabolism; and V, Organismal systems. The Y-ordinate represents the name of the KEGG metabolic pathway, and the X-ordinate represents the number of genes annotated to this pathway and their proportion to the total number of genes annotated. **(B)** Key KEGG pathway analysis of low-temperature storage of pollen in pitaya. KEGG pathway analysis depends on the transcriptomics data of pollen at different dates (0, 1, 2, and 3) stored at low temperatures. The Y-ordinate represents the transformation of the *p*-value, which was the statistical significance level of the KEGG pathway analysis, and the X-ordinate represents the importance of the pathway in the KEGG pathway analysis. Three key KEGG pathways were identified, including isoquinoline alkaloid biosynthesis, tyrosine metabolism, alanine, aspartate, and glutamate metabolism. Different colors stand for the importance of the pathway in low-temperature storage of pollen, white, yellow, and red means weak, medium, and strong, respectively.

According to the KEGG pathways annotation (Figure 6A), genes were divided into five branches, including cellular processes, environmental information processing, genetic information processing, metabolism, and organismal systems. Metabolism and organismal systems annotated the most metabolic pathways, including 12 and 10, respectively. Environmental information processing involved in only two pathways, which were signal transduction and membrane transport.

According to Figure 6B, KEGG pathway analysis showed that isoquinoline alkaloid biosynthesis, tyrosine metabolism, alanine, aspartate, and glutamate metabolism accounted for more weight among the pathways, which means that these pathways affected the low-temperature storage ability of pollen significantly.

**Figure 7:**
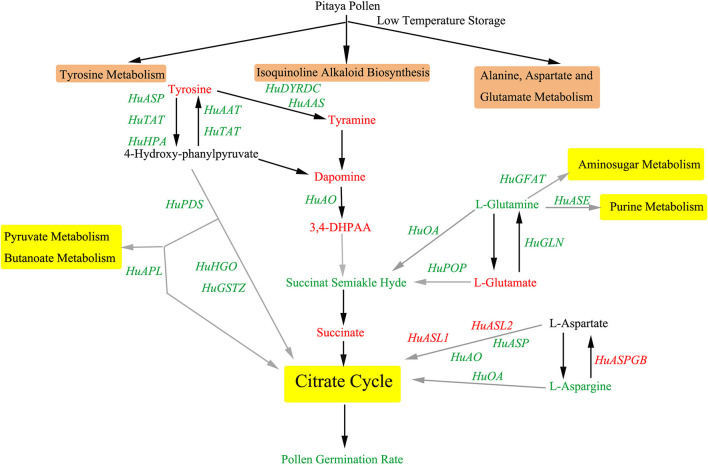
Key metabolic and transcriptional regulatory networks involved in pollen cryopreservation in pitaya. Three key KEGG pathways are shown in the brown text box, including isoquinoline alkaloid biosynthesis, tyrosine metabolism, alanine, aspartate, and glutamate metabolism. Downstream pathways of which are shown in the yellow text box. Metabolites and genes annotated in these three key pathways, black arrows mean direct pathway between the metabolites, the gray ones are brief pathways between the metabolites because some metabolites along the pathway were not detected. The changing patterns of the metabolites and genes were set in different colors, namely, red means upregulation or increase, and green means downregulation or decrease. TCA, Tricarboxylic acid cycle.

The isoquinoline alkaloid biosynthesis is involved in the biosynthesis of some important alkaloids. Through this pathway, 5 genes and 4 metabolites were detected. The contents of L-tyrosine, tyramine, dopamine, and 3,4-DHPAA increased markedly, while the gene expressions of *HuAAT, HuTAT, HuTYRDC, HuAAS*, and *HuAO* were downregulated (Figure 7). It could be implied that isoquinoline alkaloid biosynthesis disorder was significantly correlated with the decrease in pollen germination.

Tyrosine is an aromatic amino acid that plays an important role in human growth and metabolism. It is also a precursor of many metabolites in plants. Tyrosine metabolism played an important role in pitaya pollen storage. During pollen storage, the contents of tyrosine, 3, 4-dihydroyxphenylacetaldehyde, dopamine, and succinate increased significantly, and succinate semialdehyde reduced. As for the related genes, *HuTYRDC, HuAAS, HuAO, HuASP, HuTAT, HuHPA, HuPPTM, HuPDS, HuHGO, HuGSTZ* were downregulated (Figure 7), which could indicate that tyrosine metabolism pathway was inhibited during the pollen storage period, so that the content of its downstream products (including tocopherols, plastoquinone, and ubiquinone) reduced, finally caused REDOX system disorder and free radical accumulation.

As for alanine, aspartate, and glutamate metabolism (Figure 7), the content of L-glutamate increased while pyruvate, succinate semialdehyde, and L-asparagine decreased significantly. In the identified genes, only *HuASPGB, HuASL1*, and *HuGDH2* were upregulated markedly, while the other genes were downregulated during pollen storage.

### Correlation Analysis on Pollen Response to Low-Temperature in Pitaya

**Figure 8:**
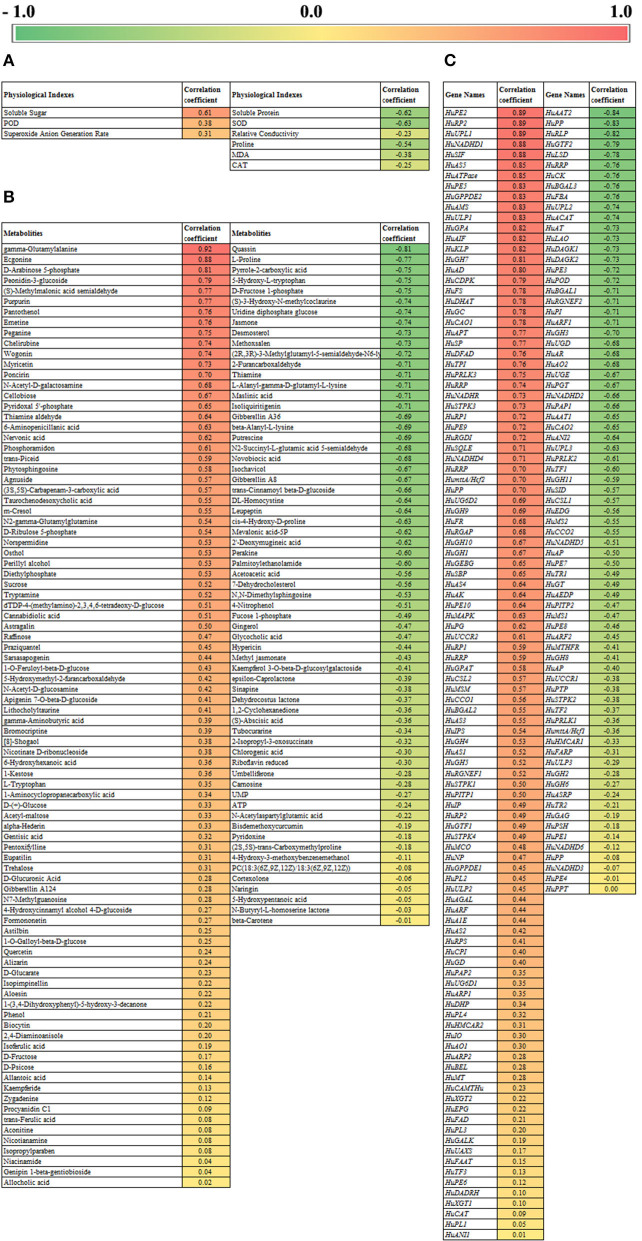
Correlation analysis of pollen germination rate with physical and chemical index **(A)**, metabolomics **(B)**, and transcriptomics **(C)**.

The results of the correlation analysis are shown in Figure 8. Figure 8A shows the correlation analysis between physiological parameters and pollen germination rate. Soluble sugar content was significantly positively correlated with pollen germination rate, with a correlation coefficient of 0.61, while soluble protein content and SOD enzyme activity were negatively correlated with pollen germination rate, with a correlation coefficient of −0.63 and −0.63. Correlation analysis between metabolomics data and pollen germination rate showed (Figure 8B) that gamma-glutamylalanine and ecgonine had the highest positive correlation coefficient with pollen germination rate (0.91 and 0.88). The correlation coefficients of D-arabinose 5-phosphate (A5P) and peonidin-3-glucoside were 0.81 and 0.79, while quassin (−0.81) and L-proline (−0.77) showed significant negative correlations. A5P is the first intermediate of the 2-keto-3-deoxy-octanoic acid (KDO) biosynthetic pathway, which is of great importance in the metabolism of sugars in microorganisms and plants. According to Figure 8C, correlation analysis between transcriptomic data and pollen germination rate showed that *HuPE2, HuRP2*, and *HuUPL1* had higher correlation coefficients of 0.89, followed by *HuNADHD1* and *HuSIF* with correlation coefficients of 0.88. For the negative correlation genes, *HuAAT2* (−0.84), *HuPP* (−0.83), and *HuRLP* (−0.82) were observed as the most significant negative correlations. These genes could be used as markers to measure pollen germination.

## Discussion

During the pollen storage, the content of soluble sugar and proline increased significantly on day 1 and day 2, respectively. A previous study indicated that increasing the contents of sugar and proline could improve the stress resistance in plants (Zhao et al., 2021). So it could be implied that low-temperature storage improved the stress resistance of pollen grain. A previous study indicated that the main components of a pollen grain tube wall were arabinose, galactose, glucose, and uronic acid, while the pollen tube wall was made mostly of glucose (Nakamura and Suzuki, 1981). It could be implied that A5P is related to the formation of the cell wall and pollen germination. So the content of sugars should be regulated and controlled at a suitable level. Furthermore, the contents of glucose and proline increased while pollen germination went down during storage, so the contents of glucose and proline could be used as indicators of pollen activity. It is reported that carbohydrate-based extract enhanced pollen germination and pollen tube growth, which means that soluble sugar played an important role in pollen growth progress (Laggoun et al., 2021). According to the correlation analyses, A5P and peonidin-3-glucoside showed high positive correlations with the pollen germination. Therefore, A5P and peonidin-3-glucoside showed potential for pollen storage in pitaya. A5P is the first intermediate of the KDO biosynthetic pathway, which is of great importance in the metabolism of sugars in microorganisms and plants, and KDO is also found in pectin polysaccharides of higher plants and algae, mainly involved in the formation of the plant cell wall (Edashige and Ishii, 1998; Becker et al., 2001; Tzeng et al., 2002). To sum up, A5P is an important compound for pollen tube formation and pollen germination in pitaya.

The relative conductivity is also an important parameter to estimate the physiological status of the pollen grain, which was generated by different ion species traversing the plant, inducing systemic responses (Bodale et al., 2021). As the relative conductivity increased rapidly on day 2 and day 3, it meant that pollen grain aged rapidly in the last 2 days. Changes in SOD and CAT activities were used as stress indicators (Etinba-Gen et al., 2019), so the ROS system changes significantly when plants suffer from stress, and the pollen grain stored at low temperature suffered from ROS disorder because of free radical accumulation. According to the correlation analyses, SOD showed a significantly negative correlation with pollen germination, implying that SOD is a vital component for the ROS system when the pollen is in a good condition, and we should focus on the activity of SOD when we store the pitaya pollen. It was reported that flavonols reduced ROS accumulation and inhibition of pollen tube growth (Muhlemann et al., 2018). As shown in Figure 3, quercetin, kaempferide, astragalin, myricetin, isoliquiritigenin, and eupatilin decreased obviously, and myricetin showed the highest positive correlation with the pollen germination among the flavonols. Therefore we can imply the decrease in myricetin and the accumulation of ROS during storage associated with the lower pollen germination. So myricetin could be used as an effective component in pollen preservation of pitaya.

As shown in Figure 8, *HuNADHD1, HuSIF, HuPE2, HuRP2*, and *HuUPL1* were positively correlated with pollen germination rate, while *HuAAT2* and *HuPP* were significantly negatively correlated. Previous studies reported that protein modification regulated the cold tolerance in plants, such as phosphorylation of *ICE1* (Ding et al., 2015), MAPK cascade (Zhang et al., 2017), and ubiquitin (Jiang et al., 2017). It can be inferred that a decrease in protein modification-related gene expressions correlated to the decrease in pollen cold tolerance and led to the loss of pollen germination. *HuRP2, HuUPL1*, and *HuAAT2* belong to the genes related to protein modification, which were significantly related to the pollen germination rate, and can be used as the genetic indicators for determining pollen activity in pitaya. At the same time, the low expression of REDOX reaction-related genes caused the accumulation of free radicals and oxidation-reduction system disorder and led to a decrease in pollen viability (Muhlemann et al., 2018).

In this study, we have found that the contents of A5P and myricetin and the gene expression patterns of *HuRP2, HuUPL1*, and *HuAAT2* had significant effects on the pollen germination in pitaya. In the future, we will focus on pollen storage technology in pitaya, mainly depending on the important metabolites related to pollen germination, which are valuable compounds for preservation agents.

## Conclusion

The pollen was still in good condition 1 day after low-temperature storage in pitaya. During low-temperature storage, the contents of soluble sugar, protein, and flavones of pollen decreased, while free radicals accumulated excessively due to the disturbance of the oxidase system, and the expression of genes related to sugar metabolism, protein metabolism, energy metabolism, and redox system was downregulated, especially the gene expression patterns of *HuRP2, HuUPL1*, and *HuAAT2* had significant effects on the pollen germination, leading to low nutrition and low energy of pollen, and eventually pollen germination was inhibited. A5P and myricetin were positively correlated with pollen germination rate, which are valuable preservation agents.

## Data Availability Statement

The datasets presented in this study can be found in online repositories. The names of the repository/repositories and accession number(s) can be found below: NCBI, PRJNA822018, and MetaboLights, MTBLS4641.

## Author Contributions

Q-mS conceived and supervised the experiments. H-fD performed the experiments. BJ and J-sZ analyzed the data, with H-fD as the co-author. J-cL sampled and carried out Q-PCR technical aspects. All authors participated in writing and have read and approved the final version of the manuscript.

## Funding

This work was supported by Project of Collaborative Innovation Center of GDAAS (XTXM202203), Science and Technology Program of Guangzhou (Grant Nos. KTP20210078 and 2014B020202010), Department of Agriculture and Rural Affairs Program of Guangdong Province (grant nos. 2020KJ257, 2019KJ116, Yue Nongji (2018) 37 and Yue Caiji (2019) 73), and Guangzhou Municipal and Technology Bureau (20212100031).

## Conflict of Interest

The authors declare that the research was conducted in the absence of any commercial or financial relationships that could be construed as a potential conflict of interest.

## Publisher's Note

All claims expressed in this article are solely those of the authors and do not necessarily represent those of their affiliated organizations, or those of the publisher, the editors and the reviewers. Any product that may be evaluated in this article, or claim that may be made by its manufacturer, is not guaranteed or endorsed by the publisher.
